# CHILDSTAR: CHIldren Living With Diabetes See and Thrive with AI Review

**DOI:** 10.1177/11795514231203867

**Published:** 2023-10-09

**Authors:** Katie Curran, Noelle Whitestone, Bedowra Zabeen, Munir Ahmed, Lutful Husain, Mohammed Alauddin, Mohammad Awlad Hossain, Jennifer L Patnaik, Gabriella Lanoutee, David Hunter Cherwek, Nathan Congdon, Tunde Peto, Nicolas Jaccard

**Affiliations:** 1 Centre for Public Health, Queens University Belfast, Belfast, UK; 2 Orbis International, New York, NY, USA; 3 Department of Paediatrics, Life for a Child & Changing Diabetes in Children Programme, Bangladesh Institute of Research & Rehabilitation in Diabetes, Endocrine & Metabolic Disorders (BIRDEM), Diabetic Association of Bangladesh, Dhaka, Bangladesh; 4 Orbis Bangladesh, Dhaka, Bangladesh; 5 Department of Ophthalmology, University of Colorado School of Medicine, Aurora, CO, USA; 6 Zhongshan Ophthalmic Center, Sun Yat-sen University, Guangzhou, China

**Keywords:** Artificial intelligence, diabetic retinopathy screening, children, young adults, diabetes

## Abstract

**Background:**

Artificial intelligence (AI) appears capable of detecting diabetic retinopathy (DR) with a high degree of accuracy in adults; however, there are few studies in children and young adults.

**Methods:**

Children and young adults (3-26 years) with type 1 diabetes mellitus (T1DM) or type 2 diabetes mellitus (T2DM) were screened at the Dhaka BIRDEM-2 hospital, Bangladesh. All gradable fundus images were uploaded to Cybersight AI for interpretation. Two main outcomes were considered at a patient level: 1) Any DR, defined as mild non-proliferative diabetic retinopathy (NPDR or more severe; and 2) Referable DR, defined as moderate NPDR or more severe. Diagnostic test performance comparing Orbis International’s Cybersight AI with the reference standard, a fully qualified optometrist certified in DR grading, was assessed using the Matthews correlation coefficient (MCC), area under the receiver operating characteristic curve (AUC-ROC), area under the precision-recall curve (AUC-PR), sensitivity, specificity, positive and negative predictive values.

**Results:**

Among 1274 participants (53.1% female, mean age 16.7 years), 19.4% (n = 247) had any DR according to AI. For referable DR, 2.35% (n = 30) were detected by AI. The sensitivity and specificity of AI for any DR were 75.5% (CI 69.7-81.3%) and 91.8% (CI 90.2-93.5%) respectively, and for referable DR, these values were 84.2% (CI 67.8-100%) and 98.9% (CI 98.3%-99.5%). The MCC, AUC-ROC and the AUC-PR for referable DR were 63.4, 91.2 and 76.2% respectively. AI was most successful in accurately classifying younger children with shorter duration of diabetes.

**Conclusions:**

Cybersight AI accurately detected any DR and referable DR among children and young adults, despite its algorithms having been trained on adults. The observed high specificity is particularly important to avoid over-referral in low-resource settings. AI may be an effective tool to reduce demands on scarce physician resources for the care of children with diabetes in low-resource settings.

## Introduction

Diabetes mellitus (DM) is one of the largest global health threats of the 21st century.^1^ The prevalence of DM has been rising more rapidly in low- and middle-income countries (LMICs) compared to high-income countries.^1^ The International Diabetes Federation (IDF) reports that 3 out of 4 people living with DM dwell in LMICs.^1^ A particularly concerning and less-studied facet of the global DM epidemic is the rapidly growing prevalence among children and young adults. Globally, an estimated 1.1 million children and adolescents (<20 years) are living with type 1 DM (T1DM).^1^ In countries with poor health systems and limited access to insulin, complications are common in young people with T1DM.^1^ Additionally, lifestyle changes across both high and low-income countries have led to a rise in obesity, and consequently type 2 DM (T2DM) among children and young adults.^2^ The World Health Organization (WHO) reports that until recently, T2DM was only seen in adults, but is now occurring with increasing frequency in children.^3^ According to IDF estimates in 2020, around 44 million children and adolescents (0-19 years) had T2DM globally.^1^ The prevalence of T2DM among children and adolescents varies across countries and regions;, however, it is more prevalent in regions with a high burden of obesity and lifestyle related risk factors.^1,4^ There is a lack of information on T1DM/T2DM, particularly in LMICs, where incidence remains largely unknown, further research and diabetes registries are required to address these gaps.

Bangladesh is a LMIC in South Asia with one of the fastest-growing economies globally.^5^ Unfortunately, the prevalence of DM is showing the largest increases in countries with high economic growth.^6^ Zabeen et al^7^ reported that the incidence of T2DM per 100 000 young people in Bangladesh aged <20 years nearly tripled from 0.2 to 0.57 between 2011 and 2018, a 12% increase per year (*P* < .001). This is particularly concerning given that early-onset T2DM exhibits accelerated development of systemic complications.^8^

Diabetic retinopathy (DR) is one of the most common microvascular complications of DM, and can lead to vision impairment and blindness if not detected and treated early.^9^ Regular DR screening and treatment reduces severe vision loss up to 90%.^10^ While the prevalence of DR among youths with well-controlled T1DM is low, adolescents with T2DM have a higher risk of DR progression compared to adults, especially when glycaemic control is poor.^11,12^ Adolescence is a critical period for the development and management of DM, as hormonal changes can complicate glycaemic control. Adolescents with DM face unique psychological challenges during puberty, including body image concerns, peer pressure and increased responsibility for DM self-management. These factors can contribute to non-adherence to treatment regimens, leading to suboptimal glycaemic control and increased risk of complications including DR.^13-15^ Regardless of their current DR status, all children and young adults with DM require regular eye examinations and education.

Artificial intelligence (AI) has emerged as a promising tool for the automated detection and screening of DR, offering the potential to improve access, efficiency and accuracy of diagnosis.^16^ AI algorithms have been trained on large datasets of adult retinal images, achieving high sensitivity and specificity. They can identify subtle changes indicative of DR in the early stage, facilitating timely intervention and preventing progression to advanced stages. However, there are few studies assessing children’s retinal images.^17^ AI offers the opportunity to shift the burden of DR screening away from physicians, especially important in LMICs where human resources are limited. Orbis International’s Cybersight AI provides automated recommendations for DR staging from fundus images based on Deep Learning algorithms trained using data from adult patients.^18^ The Diabetic Association of Bangladesh (BADAS) was established in 1956 and provides a comprehensive model of care for children and young adults with DM through programs such as Life for a Child (LFAC) and Changing Diabetes in Children (CDIC) since 2010.^19^ Orbis International has collaborated with BADAS to incorporate a scalable model of DR screening into existing child-focused models of DM care in Bangladesh.^20^ The current retrospective study analyses data from the Orbis/BADAS programme to assess the performance of the Cybersight AI system in detecting any DR and referable DR in fundus images of children and young adults, compared with the reference standard, a fully qualified optometrist certified in DR grading.

## Methods

### Selection of study participants

#### Inclusion criteria

- Children and young adults aged 3 to 26 years with DM
- Patients with gradable, macular-centred fundus images

#### Exclusion criteria

- Ungradable fundus images
- Patients with no macular-centred fundus images

Children and young adults aged 3 to 26 years with T1DM or T2DM were screened at the Dhaka BIRDEM-2 hospital, Bangladesh, as part of Orbis’ child-focused DR project, funded by United States Agency for International Development (USAID). This age range was agreed upon with the research team, as this age-range supports a seamless transition from paediatric to adult care. HbA1c, DM duration and other demographic information such as age and sex were collected. Two fundus images in each eye, 1 centred on the macula and 1 on the optic nerve, were captured by trained technicians using a digital non-mydriatic camera (Canon CR2-AF, Canon Medical Systems, Tokyo, Japan). All images were captured in a darkened room. All anonymised fundus images were transferred to the Ophthalmic Reading Centre at QUB and stored on a password-protected server. Grading was carried out retrospectively, based on the International Clinical Diabetic Retinopathy (ICDR) Scale.^21^

The reference standard was a fully qualified optometrist (KC), certified in DR grading through the Gloucestershire Retinal Education Group’s Diabetic Retinopathy Grading Course.^22^ A subset of images (20%) selected by stratified random sampling were re-graded by an external senior DR grader (RB). Any disagreements were discussed with any required adjudication provided by a retina specialist and clinical lead for Diabetic Eye Screening in Northern Ireland (TP). This additional subset of images was also reviewed for inner retinal sheen, which is common in paediatric fundus images. It was suspected that this finding might affect the performance of the AI software, which had been trained only using adult images, therefore the graders notated if sheen was present or not present. Patients were originally screened and reviewed by a clinician to maintain patient safety; therefore, fundus images were re-graded in this study for research purposes only.

Subsequently, all gradable fundus images, anonymised with a unique patient registration number, were uploaded to Orbis International’s Cybersight AI (New York, USA) for interpretation. Cybersight AI is available free of charge to eye health professionals in LMICs and is accessible upon completion of registration on Orbis International’s telehealth platform, Cybersight.^23^ Patient encounters that included ungradable images (as assessed by graders) were excluded from this analysis (37 patient encounters excluded). AI results were based only on macula-centred images and patient encounters with missing macula-centred images were excluded from this analysis (21 patient encounters excluded). When multiple macula-centred images were available for a given eye and clinical encounter, the 1 producing the highest AI output was used for analysis. Two main outcomes were considered at a patient level:

- Any DR, which was defined to include mild non-proliferative diabetic retinopathy (NPDR), moderate NPDR, severe NPDR, proliferative DR and quiescent PDR.
- Referable DR, which was defined to include moderate NPDR, severe NPDR, proliferative DR and quiescent PDR.

The machine learning model used in this study is based on the EfficientNet-B3 Convolutional Neural Network (CNN) architecture.^24^ Training of the model was carried out prior to the study using the *fastai* library^25^ based on independent datasets with no data originating from Bangladesh. A pretrained EfficientNet-B3 model was fine-tuned on a regression task that consisted of predicting a continuous variable between 0 and 4, encoding the 5 categories of the ICDR Severity Scale (normal, mild NPDR, moderate NPDR, severe NPDR and proliferative DR). To improve generalisation to unseen images, data augmentation techniques such as random zoom, flipping and rotation were applied during training. The input to the CNN was a single or batch of macula-centred fundus photograph(s). Preprocessing included: (i) resizing images to 512 × 512 pixels with preserved aspect ratio and (ii) normalisation of image pixel values based on mean and standard deviation of pixel values in the pretraining dataset. The output was a continuous value, ranging from 0 (normal) to 4 (most severely affected), which was subsequently thresholded to obtain a binary outcome for *Any DR* and *Referable DR* (cutoff values of 0.45 and 1.9, respectively). Those values were obtained through evaluation of the model on independent, validation datasets.

### Statistical methods

Grading data were transferred to SPSS Statistics version 25.0 software (IBM, Armonk, NY) and SAS version 9.4 (Cary, NC) for analysis. Agreement analyses were conducted to assess the grading repeatability as an index of reliability. Diagnostic test performance comparing Orbis International’s Cybersight AI with the reference standard human DR grader was assessed using the Matthews correlation coefficient (MCC), area under the receiver operating characteristic curve (AUC-ROC), area under the precision-recall curve, Kappa, sensitivity, specificity, positive predicative values (PPV) and negative predictive values (NPV). Estimates of each and corresponding 95% confidence intervals are presented. Potential predictors of the AI system correctly identifying referable DR versus normal/non-referable disease were assessed with univariate and multivariable logistic regression models that included gender, severity of DR, age of participant and duration of DM.

## Results

**Figure 1.**
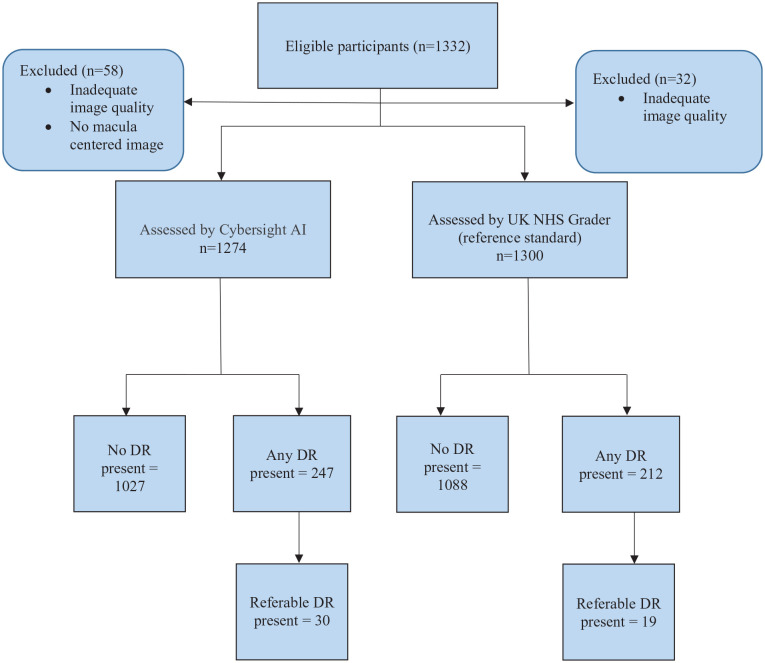
Flow diagram to illustrate enrolment of participants included in the study.

Among 1332 participants, 32 (2.40%) had images of inadequate quality for grading and 26 (1.95%) did not include macula-centred images. Among 1,274 participants (53.1% female, mean age 16.7 ± 4.60 years) with fundus images interpreted by the AI, 19.4% (n = 247) had any DR present according to the AI, as did 16.6% (n = 212) according to the reference standard. For referable DR, 2.35% (n = 30) were detected by AI and 1.49% (n = 19) by the reference standard (Figure 1). The reference standard was masked to the AI grading and vice versa. Out of a random sample of 289 participants, 97.0% were assessed by the standard human graders to have sheen present in 1 (macular-centred) or more of their fundus photographs. The AI system did not use any specific image enhancement and/or pre-processing to reduce retinal sheen observed in younger humans.

The mean duration of DM was 4.90 years (SD 3.68 years) and mean HbA1c was 9.48% (SD 2.21%) among the 686 participants (53.8%) with such data. The mean age for participants with any DR, was 20.2 (SD 3.53) years according to the AI and 20.2 (SD 3.46) years according to the reference standard, while for referable DR mean ages were 22.5 (SD 2.86) years and 21.3 (SD 3.23) years for the AI and reference standard respectively (Table 1).

**Table 1.** Demographic and clinical characteristics of participants.

	All participants	Any DR present		Referable DR present	
		According to AI	According to certified NHS grader	According to AI	According to certified NHS grader
Patients, number (row %)	1,274	247 (19.4%)	212 (16.6%)	30 (2.35%)	19 (1.49%)
Female, number (column %)	676 (53.1%)	135 (54.7%)	106 (50.0%)	10 (47.6%)	8 (42.1%)
Age (years), mean ± SD	16.7 (4.60)	20.2 (3.53)	20.2 (3.46)	22.5 (2.86)	21.3 (3.23)
<5	13 (1.02%)	0	1	0	0
6-10	111 (8.71%)	2	0	0	0
11-15	351 (27.6%)	23	17	1	1
16-18	336 (26.4%)	53	47	1	3
⩾19	463 (36.3%)	169	147	28	15
Diabetes duration (years), (mean ± SD)^a^	4.90 (3.68)	7.07 (3.99)	7.39 (4.12)	9.54 (4.90)	9.48 (5.04)
HbA1c (%)	686	110	101	14	10
(mean ± SD)	9.48 (2.21)	9.58 (2.48)	9.55 (2.56)	11.3 (1.90)	10.2 (2.41)
<7%, number (%)	78 (11.4%)	15 (13.6%)	15 (14.8%)	0 (0%)	1 (10.0%)
⩾7%, number (%)	608 (88.6%)	95 (83.4%)	86 (85.2%)	14 (100%)	9 (90.0%)

Abbreviations: AI, artificial intelligence; NHS, National Health System; SD, standard deviation.

a Two patients with missing or incorrect date of birth or date of diagnosis are not included in diabetes duration.

The sensitivity and specificity of AI to detect any DR compared to the reference standard were 75.5% (95% CI: 69.7-81.3%) and 91.8% (95% CI: 90.2 93.5%), while for referable DR the figures were 84.2% (CI: 67.8-100%) and 98.9% (CI: 98.3-99.5%). The PPV values were 64.8% (95% CI: 58.8-70.7%) and 53.3% (95% CI: 35.5-71.2%) for any and for referable DR, while corresponding NPV values were 94.9% (93.6-96.3%) and 99.8% (99.5-100%) (Table 2).

**Table 2.** Performance of the artificial intelligence system compared to the certified NHS human grader (reference standard).

	Any DR	Referable DR
Present	212	30
Absent	1062	1244
Matthews correlation coefficient (MCC)	63.4%	66.4%
Area under the ROC curve (95% CI)	91.2%	97.6%
Area under the precision-recall curve (95% CI)	76.2%	76.5%
Kappa (95% CI)	63.1% (57.5-68.7%)	64.7% (49.0-80.3%)
Sensitivity (%) (95% CI)	75.5% (69.7-81.3%)	84.2% (67.8-100%)
Specificity (%) (95% CI)	91.8% (90.2-93.5%)	98.9% (98.3-99.5%)
Positive predictive value (%) (95% CI)	64.8% (58.8-70.7%)	53.3% (35.5-71.2%)
Negative predictive value (%) (95% CI)	94.9% (93.6-96.3%)	99.8% (99.5-100%)

The sensitivity and specificity of the AI at eye level was also calculated, and these rates were similar to the presented patient-level measures with slightly lower sensitivities and higher specificities (72.1 and 95.2%, respectively for any DR, and 74.2 and 99.6% for referable DR).

Potential predictors for AI to correctly identify referable DR are highlighted in Table 3. Children in whom the AI made correct diagnosis were younger (Mean age 16.7 years, SD = 4.56 vs 22.9 years, SD = 3.12, *P* < .0001) and had a shorter duration of DM (Mean = 4.85 years, SD = 3.64 vs 8.50 years, SD = 4.90, *P* < .0001). Gender was not associated with correctly identifying referable DR in univariate (*P* = .328) or multivariate regression (*P* = .542) (Table 3).

**Table 3.** Odds ratios and confidence intervals of potential predictors of correct AI grade for referable DR.

	Referable DR		Referable DR	
	Correct	Incorrect	Univariate OR (95% CI)	Multivariate OR (95% CI)
			*P*-value	*P*-value
	1257 (98.7%)	17 (1.33%)	**–**	**–**
Gender				
Male	588 (98.3%)	10 (1.67%)	0.62 (0.23-1.63)	0.73 (0.27-1.99)
Female	669 (99.0%)	7 (1.04%)	0.328	0.542
Severity of DR				
No DR	1,059 (99.7%)	3 (0.28%)	Reference	
Mild	182 (94.3%)	11 (5.70%)	0.05 (0.01-0.17)	
Moderate+	16 (84.2%)	3 (15.8%)	0.02 (0.003-0.08)	
	Mean (SD)	Mean (SD)		
Age (years)	16.7 (4.56)	22.9 (3.12)	0.65 (0.54-0.76)	0.66 (0.56-0.80)
			<.0001	<.0001
Diabetes duration (years)	4.85 (3.64)	8.50 (4.90)	0.82 (0.75-0.91)	0.95 (0.84-1.08)
			<.0001	.422

## Discussion

To the best of our knowledge, this is the second study to explore the performance of an AI system to detect DR in a population of children and young adults.^17^ and is the first to do so in a LMIC. Use of fundus photography to diagnose DR in youth was previously supported by Strul et al^26^ where telemedicine was implemented to review fundus photographs and showed greater detection of DR compared to standard ophthalmic examinations. Wolf et al^17^ assessed the performance of an AI system to detect more than mild DR in a paediatric population in a high-income country, USA. While both AI systems were trained using adult images, they both reported high diagnosability rates and conclude that AI is a safe and effective tool for detecting referable DR in children and young adults.^17,18^

Early onset T2DM among both children and adults is associated with even higher rates of retinopathy (and related complications) than T1DM of the same duration.^27,28^ This is likely related to the greater number of risk factors for retinal damage present in T2DM patients, such as obesity, high blood pressure and abnormal lipid levels. Maintaining optimal glycaemic control, as reflected by HbA1c levels, is crucial for preventing DR. Lowering HbA1c levels has been associated with reduced risk and DR progression in both T1DM and T2DM. Regular self-monitoring of blood glucose levels is crucial in managing DM and preventing DR, allowing individuals to make timely adjustments to their treatment plans to prevent complications.^12,29-31^ Furthermore, longer duration of DM is strongly associated with the development and progression of DR.^30-32^

The prevalence of DR in children and young adults is low, nonetheless, it is not zero, and so monitoring is needed. It is important to train AI algorithms to accurately identify normal cases in order to avoid overwhelming healthcare systems with false positives. It is thus encouraging that this study found high specificity in the detection of referable DR at 98.9% and comparable performance to other AI systems interpreting adult images when evaluated in actual clinical workflows rather than in lab-based evaluations.^16^ Sensitivity in our study was also comparable to that reported by Wolf et al^17^ for DR in children and young people. Given the relatively low prevalence, the generally good accuracy of AI screening in our study and the very limited availability of persons trained to carry out diabetic eye examinations on children in this setting, our results suggest that AI approaches such as the one tested here are ideally suited to assist in the cost-effective ocular screening of children living with DM in LMICs.

Zabeen et al^33^ reported the prevalence of DR among children, adolescents and young adults in Bangladesh is 6.6%, with the majority having mild non-proliferative DR. This study found a higher prevalence of any DR as detected by both AI and the reference standard of 19.4 and 16.6% respectively. However, referable DR was found in only 2.35% of participants according to AI, and 1.49% according to the reference standard. The prevalence of DR among children and adolescents in previous studies ranged from 4 to 13.7%.^12,17,33^ The low prevalence of DR among youths supports AI as a DR screening strategy in this LMIC setting, where having scarce trained experts examine many normal children will not be a desirable use of resources.^35^ This is supported by economic modelling studies, which show both cost and workload savings when automated DR screening models are implemented with simple grading measures of disease/no disease as the outcome.^36^

Consistent the high specificity of the AI, modelling shows the AI performs very well for participants with mild or no DR. Sheen was present in the majority of images within the dataset, but encouragingly did not appear to lead to many false positive grades, as have been reported in other AI studies where sheen reflections were mistaken as exudates.^17,34^ Further exploration of sheen severity in future studies is required to assess its true impact on DR grading. Human graders had access to 2 fields-of-view per eye; however, it is common for AI solutions to screen solely based on macula-centred images. A single image (macular-centred) is also used in some successful DR screening programmes, such as the Scottish DR screening programme.^37^

As suggested by Liu et al^6^ the growing burden of DR screening cannot be adequately addressed by eye hospitals alone, and lack of infrastructure and human resources are the main barriers to DR screening in LMICs.^35^ One of the benefits of using AI for DR screening is that screening programs can be implemented outside of eye hospitals and in locations already routinely accessed by patients with DM, such as endocrinology or primary care clinics. Multiple studies have demonstrated the feasibility of AI DR screening programs at such settings for adults with DM.^38-41^ This strategy is all the more appealing for children and young adults, among whom there is documented low compliance with routine diabetic eye screenings^17^ and additional demand on caretakers’ time. Increased adherence to DR referrals when AI is implemented as a screening tool was recently demonstrated by Mathenge et al^42^ in Rwanda and also by Wolf et al^17^ in the paediatric setting. In a separate study, Wolf et al^43^ also showed the cost-effectiveness of paediatric DR screening using autonomous AI. All this suggests that AI is an effective tool for DR screening in young people, especially in low-resource settings such as Bangladesh.

Strengths of the study include the larger sample size relative to previously published studies^17,25^ and the focus on LMICs, where the highest burden of DM and its associated complications exist.^1^ Limitations include missing/incomplete variables such as, HbA1c, DM type, methods of blood glucose monitoring, for analysing factors predicting accurate AI, and the retrospective design. Given the high proportion of normal images, expected in this young cohort, MCC and the PR-AUC were used as they are less sensitive to class imbalances. There were differences in grading methods as the AI system uses only macula images, and the human graders used both macula and disc-centred. In addition, power calculations to estimate the sample size needed were not performed a priori. Also of note, the analysis was done using the existing version of the DR algorithm, with an updated version expected in the near term. Additional prospective studies and pragmatic trials are needed to broaden the evidence base for use of AI in paediatric DR screening.

## Conclusions

Cybersight AI performed well on fundus images from children and young adults despite its algorithms being trained on adult eyes. The high specificity is particularly important for children, where the majority of images are likely to be normal. AI may be an effective tool to screen children with DM to identify referable DR, and could help to reduce demands on scarce physician resources in low-resource settings.

## Declarations

**Ethics approval and consent to participate:** This study was conducted in accordance with the principles of the Declaration of Helsinki. Ethical review for capturing imaging data was obtained (Ethics reference No: Southasia-hrec-2020-1-03) Ethical review for secondary image analysis was also carried out by the Asian Institute of Disability and Development (AIDD) Research Committee in Dhaka (Ethics Reference No: Southasia-hrec-2020-8-01). Verbal consent was obtained from participants and their guardians as necessary and approved by the Research Ethics Committee.

**Consent for publication:** Not applicable.

**Availability of data and materials:** All data relevant to the study are included in the article or uploaded as supplementary information.
